# Immunohistochemistry Successfully Uncovers Intratumoral Heterogeneity and Widespread Co-Losses of Chromatin Regulators in Clear Cell Renal Cell Carcinoma

**DOI:** 10.1371/journal.pone.0164554

**Published:** 2016-10-20

**Authors:** Wei Jiang, Essel Dulaimi, Karthik Devarajan, Theodore Parsons, Qiong Wang, Lili Liao, Eun-Ah Cho, Raymond O'Neill, Charalambos Solomides, Stephen C. Peiper, Joseph R. Testa, Robert Uzzo, Haifeng Yang

**Affiliations:** 1 Department of Pathology, Anatomy and Cell Biology, Thomas Jefferson University, Philadelphia, Pennsylvania, United States of America; 2 Department of Pathology, Fox Chase Cancer Center, Philadelphia, Pennsylvania, United States of America; 3 Biostatistics and Bioinformatics Facility, Fox Chase Cancer Center, Philadelphia, Pennsylvania, United States of America; 4 Cancer Biology Program, Fox Chase Cancer Center, Philadelphia, Pennsylvania, United States of America; 5 Department of Surgical Oncology, Fox Chase Cancer Center, Philadelphia, Pennsylvania, United States of America; University of Alabama at Birmingham, UNITED STATES

## Abstract

Recent studies have shown that intratumoral heterogeneity (ITH) is prevalent in clear cell renal cell carcinoma (ccRCC), based on DNA sequencing and chromosome aberration analysis of multiple regions from the same tumor. *VHL* mutations were found to be universal throughout individual tumors when it occurred (ubiquitous), while the mutations in other tumor suppressor genes tended to be detected only in parts of the tumors (subclonal). ITH has been studied mostly by DNA sequencing in limited numbers of samples, either by whole genome sequencing or by targeted sequencing. It is not known whether immunohistochemistry (IHC) can be used as a tool to study ITH. To address this question, we examined the protein expression of PBRM1, and PBRM1-related proteins such as ARID1A, SETD2, BRG1, and BRM. Altogether, 160 ccRCC (40 per stage) were used to generate a tissue microarray (TMA), with four foci from each tumor included. Loss of expression was defined as 0–5% of tumor cells with positive nuclear staining in an individual focus. We found that 49/160 (31%), 81/160 (51%), 23/160 (14%), 24/160 (15%), and 61/160 (38%) of ccRCC showed loss of expression of PBRM1, ARID1A, SETD2, BRG1, and BRM, respectively, and that IHC could successfully detect a high prevalence of ITH. Phylogenetic trees were constructed that reflected the ITH. Striking co-losses among proteins were also observed. For instance, ARID1A loss almost always accompanied PBRM1 loss, whereas BRM loss accompanied loss of BRG1, PBRM1 or ARID1A. SETD2 loss frequently occurred with loss of one or more of the other four proteins. Finally, in order to learn the impact of combined losses, we compared the tumor growth after cells acquired losses of ARID1A, PBRM1, or both in a xenograft model. The results suggest that ARID1A loss has a greater tumor-promoting effect than PBRM1 loss, indicating that xenograft analysis is a useful tool to investigate how these losses impact on tumor behavior, either alone or in combination.

## Introduction

Tumors are generally thought to originate from one or a few cancerous cells with founding mutation(s), with additional mutations occurring in later stages of tumor development to promote disease progression [1]. During this process of tumor evolution, one question arises: do the additional mutations exist in all the tumor cells, such that all progeny have identical genetic lesions, or do they occur in a subset of cells? Multiple genetic analyses have revealed that the latter is true, i.e., that different regions of the same tumor share the founding mutation(s) but have different subsequent mutations, and this regionally diverse mutational landscape is called Intratumoral Heterogeneity (ITH). ITH has been described in leukemia [2], glioblastoma [3], as well as in colon [4], pancreatic [5], ovarian [6], breast [7] and clear cell renal cell carcinoma (ccRCC) [8, 9]. This phenomenon is prevalent as it exists in primary tumors and metastatic sites, and it is also discovered in the recurrent tumors after surgical removal [9]. ITH can be defined by DNA mutations, genomic copy number changes, or changes in DNA methylation patterns [9]. The different genetic makeups of different regions suggest that, during tumor development, additional genetic changes happened in a branched fashion instead of a linear fashion, giving rise to multiple clones coexisting in the same tumor.

ITH poses a serious challenge to precision medicine. Precision medicine assumes that tumors in different patients have different genetic mutations, and the presence of specific mutations indicate sensitivities to certain treatments. Thus, treatment options must be individually tailored according to the mutation profile of each patient. Often a single biopsy is applied to assay the mutational profiles of the patients. If the tumors have regional heterogeneity, then the evaluation of a single site will likely miss many DNA mutations that are present in other regions of the same tumor and will also fail to pinpoint which mutation(s) is most prevalent and whether it should be targeted. Such incomplete information for a given tumor is likely to negatively impact the selection of therapeutic options. Effective therapeutic options might be overlooked, and wrong choice might be made.

In ccRCC, inactivation of the von-Hippel Lindau tumor suppressor gene, *VHL*, either through DNA mutations or promoter hypermethylation, is the founding tumorigenic event [10, 11]. VHL loss-of-function occurs in at least 80% of sporadic ccRCC tumors. Three large-scale sequencing studies identified additional mutations in tumor suppressors that participate in epigenetic regulation [12–14]. Approximately 40% of ccRCC tumors harbor mutations in the polybromo-1 (*PBRM1*) tumor suppressor gene, which encodes a component of a SWI/SNF chromatin-remodeling complex [12]. Additionally, 10–15% of ccRCCs have mutations in either the BRCA1-associated protein 1 gene (*BAP1*) or SET domain containing 2 gene (*SETD2*), encoding a histone deubiquitinase and a histone methyltransferase, respectively [13]. Furthermore, *PBRM1* mutations have been found to confer a slight increase of death risk, while *SETD2* or *BAP1* mutations were associated with serious death risks in ccRCC patients [15–17].

Gerlinger et al. firmly established that ITH in ccRCC was prevalent. Through exome sequencing of multiple intratumoral regions, they found that ITH preceded any treatment [9]. They also discovered that, in the same tumor, distinct and spatially separated inactivating mutations could happen to the same tumor suppressor genes, e.g., *SETD2*, *PTEN* and *KDM5C*, suggestive of convergent phylogenetic evolution. Gene-expression signatures, allelic composition and ploidy profiling analysis all revealed extensive ITH. When all the changes were compared, chromosome 3p loss and *VHL* alterations were the only ubiquitous events in ccRCC, and they were defined as truncal losses [8]. In the case of *PBRM1* mutations, 50% of tumors with *PBRM1* mutations were truncal [8]. The vast majority (>70%) of driver mutations were mapped onto branches of the phylogenetic trees, while less than one third of driver mutations in two genes were truncal mutations. According to their own estimate, if more biopsies were taken, more driver mutations would be found [9]. These ITH findings were corroborated by another group that sequenced *VHL*, *PBRM1*, *SETD2*, *BAP1* and *KDM5C* in multiple regions of the same ccRCC tumors [18]. Collectively, these studies imply that analysis of a single biopsy severely underestimates the mutational profiles of a ccRCC tumors.

To date, ITH in ccRCC has been mostly studied using Next Gen Sequencing, which revealed DNA mutations in exomes. ITH in ccRCC has also been studied based on the assessment of DNA copy number changes, gene expression or ploidy, but mutational analysis has provided the most detailed information to facilitate comparisons. While DNA sequencing provides high quality data and high resolution, the major drawback of this approach is that it is expensive and labor intensive, so the total number of the samples that are analyzed is generally low, thus lacking statistical power. Immunohistochemistry (IHC) was also used to probe the samples, but it was only used to characterize samples after ITH was identified by other techniques, not as a tool to reveal ITH in the first place.

Since IHC is a mature technology that can be applied to human sample relatively inexpensively once a tissue microarray (TMA) is built, in this study we asked whether IHC on ccRCC TMAs could be used to detect ITH. Four spatially distinct regions from each ccRCC tumor were selected for TMA. Forty cases were chosen for each tumor stage, with a total of 160 cases. We decided to stain for PBRM1, ARID1A, BRG1, BRM, and SETD2 as they represent important players in ccRCC. We report that IHC can readily detect prevalent ITH in a relatively large series ccRCC, and striking co-losses among proteins were observed. Finally, we demonstrate in xenografts of ccRCC how losses of PBRM1 and ARID1A impact tumor growth.

## Materials and Methods

### Sample preparation and TMA preparation

Written informed patient consent was obtained under a protocol (IRB#13–810) approved by the IACUC of Fox Chase Cancer Center (FCCC). All samples were collected in accordance with institutional guidelines and protocols.

Altogether, 160 ccRCC patients with available archived formalin-fixed, paraffin-embedded (FFPE) tumor tissue were identified based on a search of the FCCC kidney cancer database; 40 cases from each of the 4 stages (Stage I-IV) were randomly selected. All cases were reviewed by the same pathologist (*E*.*D*.). Four different, well-separated areas from each tumor were selected for assessment of intratumoral heterogeneity. Eight TMAs were generated by the FCCC Biosample Repository.

### IHC and scoring

Antigen retrieval was performed with the Ventana Discovery ULTRA staining platform, using Discovery CCI (Ventana cat. #950–500) for a total application time of 64 min. Primary immunostaining was performed using antibodies against PBRM1 (1:50), ARID1A (1:250), BRM (1:50), BRG1 (1:200), and SEDT2 (1:100) using Ventana Antibody Dilution Buffer (Ventana cat. #ADB250) with a 44-min incubation at room temperature. Secondary immunostaining used a rabbit Horseradish Peroxidase (HRP) multimer cocktail (Ventana cat#760–500) and immune complexes were visualized using the ultraView Universal DAB (diaminobenzidine tetrahydrochloride) Detection Kit (Ventana cat#760–500). Slides were then washed with a Tris-based reaction buffer (Ventana cat. #950–300) and stained with Hematoxylin II (Ventana cat. #790–2208) for 8 min. The antibodies used were: PBRM1 (Bethyl labs, cat. # A301-591A), ARID1A (Sigma-Aldrich, cat. # HPA005456), BRM (Sigma-Aldrich, cat. # HPA029981), BRG1 (Abcam, cat. # ab110641), and SETD2 (ProSci, cat. # 30–305).

Two pathologists (*W*.*J*., *T*.*P*.) independently performed the scoring of the immunostained TMAs. The staining of tumor foci was scored as 2 if greater than 50% of tumor cells were positive, 1 if less than 50% but greater than 5% of tumor cells were positive, and 0 if less than 5% of tumor cells were positive. In the cases where the foci had different scores, two pathologists examined these foci together to reach a consensus. For each tumor, if one marker was scored as 0 in one focus, then that tumor is also considered to have a score of 0 for that marker.

### Cell culture

Kidney cancer cell lines 786-O and Ren-02 were previously described [19, 20]. All cell lines were maintained in glutamine-containing DMEM medium supplemented with 10% fetal bovine serum (FBS) and 1% penicillin and streptomycin.

### Western blot

Cells were washed in PBS buffer before being lysed with EBC buffer (50 mM Tris, pH 8.0; 120 mM NaCl; 0.5% NP-40) supplemented with a protease inhibitor cocktail. The same total amount of each lysate was loaded and resolved by SDS–PAGE and analyzed by immunoblotting. The blots were developed with Super Signal Pico substrate (Pierce Biotechnology, Rockford, IL) or Immobilon Western substrate (Millipore, Billerica, MA). The antibodies were the same as the ones used for IHC.

### Short hairpin RNAs (shRNAs)

All the shRNA constructs except for SCR were obtained from Sigma (St Louis, MO). The sequences were: SCR: GCGCG CUUUGUAGGAUUCGTT; PBRM1-94: CCGGAGTCTTTGATCTACAAA; ARID1A-90 CCTCTCTTATACACAGCAGAT; BRM-329: GCTGAGAAACTGTCACCAAAT; BRG1-551: CCGAGGTCTGATAGTGAAGAA; SETD2-30: CCTGAAGAATGATGAGATAAT; SETD2-32: GCCCTATGACTCTCTTGGTTA.

### Nude mice xenograft analysis

All animal experiments were conducted in accordance with a protocol (#01462-935A) approved by the IACUC of Thomas Jefferson University. The subcutaneous nude-mice xenograft assay was performed as described [21]. For each cell line, 10^7^ cells were injected subcutaneously into the flanks of nude mice. Each mouse was injected with two cell lines, one on each flank. The mice were anesthetized with isoflurane before injection. The mice were monitored once weekly by an investigator from Yang lab during the experiment and daily by the staff of the animal facility. The mice were followed according to an early/humane endpoint protocol, with. euthanization of animals if the tumors become ulcerated, impede the mice’s ability to reach food and water, or cause weight loss of 20%. In our experiments, no mouse died before the experimental endpoint. The mice were maintained in groups of five per cage with 12 hours of light and 12 hour of darkness light cycle. Food and water were monitored daily by staff from the animal facility. Forty immunocompromised male nu/nu nude mice of four weeks old were purchased from Taconic or Jackson labs. All mice were sacrificed by CO_2_ inhalation 8 to 10 weeks after injection of cells, and tumors were excised and weighed.

Results are reported as mean ± s.e. of the mean. Nine to ten pairs of tumors were compared. Results were statistically evaluated with Mann–Whitney U statistic analysis from SigmaPlot.

### Statistical analysis

The two-sided Fisher’s exact test was used to test the association between variables (PBRM1 vs. ARID1A etc.) in the 2x2 tables shown in S2 Table.

## Results

### Validation of the antibodies used for IHC

**Fig 1:**
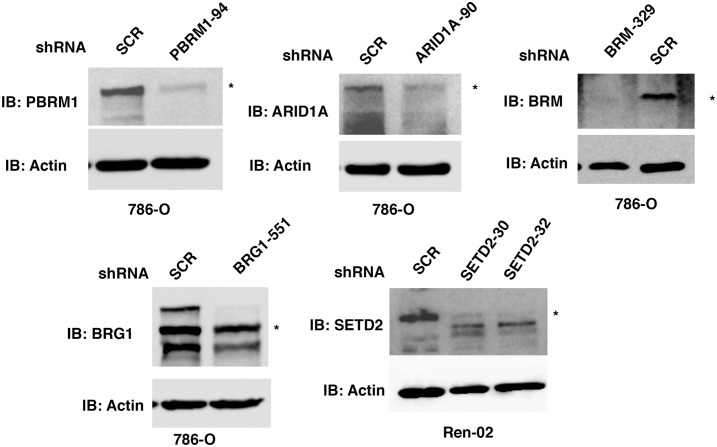
Validation of the antibodies used for IHC. Kidney cancer cell lines stably expressing control shRNA or shRNA against *ARID1A*, *PBRM1*, *BRG1*, *BRM*, and *SETD2* were used for western blot analysis with indicated antibodies.

In order to perform IHC on the TMAs of ccRCC tumor tissues, we sought to validate the specificity of each antibody we intended to use for scoring. The anti-PBRM1 antibody from Bethyl Laboratories revealed a major band of ~180 KDa which was diminished upon stable expression of a shRNA construct against *PBRM1* (Fig 1, *top left*). The same antibody was used by others for IHC and the loss of IHC signal mostly coincided with DNA mutations in *PBRM1* [17]. The anti-ARID1A antibody from Sigma-Aldrich revealed a major band of ~250 KDa which was diminished upon stable expression of a shRNA construct against *ARID1A* (Fig 1, *top middle*). This antibody has been previously used and validated by the Human Protein Atlas project [22, 23]. The anti-BRM antibody from Sigma-Aldrich detected a protein near 200 KDa, which was mostly abolished by a shRNA construct against *BRM* (Fig 1, *top left*). This antibody was also used and validated by the Human Protein Atlas project [22, 23]. It was also used for IHC in a recent publication to detect BRM in small cell carcinoma of the ovary, hypercalcemic type (SCCOHT), which revealed specific co-losses of BRG1 and BRM in these tumors [24]. The anti-BRG1 antibody detected multiple bands in the 200 KDa size range, which were diminished by a shRNA construct against *BRG1* (Fig 1, *bottom left*). This antibody was rigorously tested and confirmed by IHC staining SCCOHT tumors that contained either germline or somatic mutations in *BRG1* [24, 25]. There was a close correlation between loss of IHC staining of the tumor cells and the presence of *BRG1* mutations. The SETD2 antibody from Pro-Sci detected a band near 250 KDa, which was suppressed by two independent shRNA, constructs in the ccRCC cell line Ren-02 [20](Fig 1, *bottom right*). This antibody was shown by the supplier to detect an IHC signal in human placenta.

### Immunohistochemical analysis of ccRCC foci on TMAs

**Fig 2:**
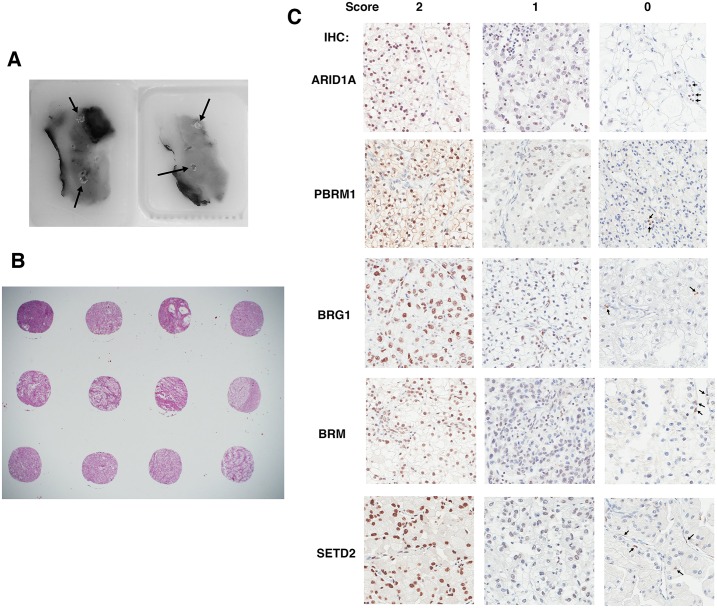
Immunohistochemical analysis of ccRCC foci from TMAs. A) Two FFPE ccRCC tissue blocks from one tumor with four cores excised (indicated by arrows). B) H&E stained foci from three tumor samples. C) Representative foci stained for different markers showing different scores. The arrows point to stromal cells that stained positive for the markers.

In order to investigate ITH, four foci from different areas from each tumor were macro-dissected to construct TMAs (Fig 2A). Except for one case where the amount of tissue was too little so only two foci were taken, each tumor was represented by four foci (Fig 2B). The antibodies validated in Fig 1 were used to stain five sets of the TMA. All five proteins were stained primarily in the nucleus (Fig 2C), which was not surprising given that each is known to be a chromatin regulator. In the case of foci that scored as 1, to be stringent we still considered them positive for expression, even though the staining was generally weak and spotty (Fig 2C). In the case of the foci that scored as 0, there was a concern that the IHC simply failed on that sample, not because the tumor cells lost protein expression. To counter that, we looked for positively stained stromal cells. In most cases scored 0, the stromal cells, but not the tumor cells, still stained positive, suggesting that the IHC was successful and indicative of loss of protein expression in tumor cells (Fig 2C, arrows indicated positively stained stromal cells). Scores for all foci are summarized in S1 Table.

### Summary of protein expression losses in tumors and foci

**Fig 3:**
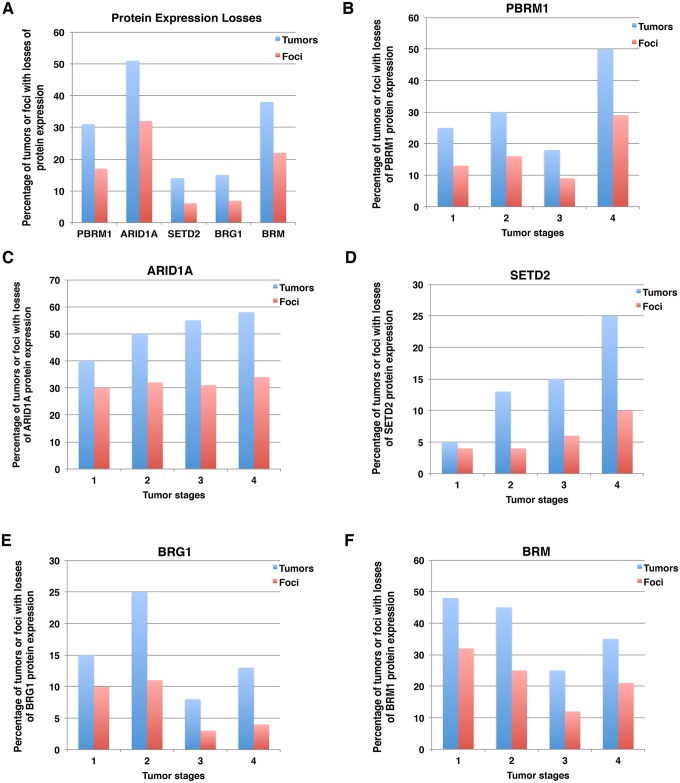
Summary of losses of expression of protein markers in ccRCC tumors and foci. A) Overall losses of each marker in tumor and foci. Loss of individual marker in different tumor stages: PBRM1 (B), ARID1A (C), SETD2 (D), BRG1 (E), BRM (F).

Usually if a DNA mutation is detected as a fraction of the wild type allele from a single biopsy, then that tumor is considered positive for that mutation. To be consistent with this criterion, we decided that if protein expression was absent in one focus out of four foci from a tumor, then that tumor would be considered to have loss of expression of that marker. After tallying protein expression losses, we found that 31% of the 160 tumors lost expression of PBRM1, 51% of them lost ARID1A, 14% lost SETD2, 15% lost expression of BRG1, and 38% lost expression of BRM (Fig 3A). Because usually not all four foci of any one tumor showed lost expression of a specific marker, the percentage of all foci that show loss of expression was lower than for all cases. Thus, for foci, lost protein expression was lower: i.e., 17%, 32%, 6.1%, 6.9% and 22% of foci lost expression of PBRM1, ARID1A, SETD2, BRG1 and BRM, respectively (Fig 3A).

For each marker, we examined protein losses in different tumor stages. For the sake of simplicity, we report the percentage of tumors with protein expression loss followed by percentage of foci with protein loss in parenthesis. For instance, 25% (13%) indicates that 25% of tumors and 13% of all foci exhibited protein expression loss of the indicated marker. PBRM1 loss tended to increase with rising tumor stage, i.e., 25% (13%) for stage I, 30% (16%) for stage II, 18% (9%) for stage III, and 50% (29%) for stage IV (Fig 3B). A similar trend was observed for ARID1A loss, with 40% (30%), 50% (32%), 55% (31%), and 58% (34%) from stages I to IV, respectively (Fig 3C). SETD2 loss showed a more dramatic increase with increasing tumor stages; 5% (4%), 13% (4%), 15% (6%), and 25% (10%) from stages I to IV, respectively (Fig 3D). BRG1 loss fluctuated among tumor stages. From stages one to four 15% (10%), 25% (11%), 8% (3%), and 13% (4%) lost BRG1 expression (Fig 3E). For BRM, there was a modest decrease in the percentage of protein loss with increasing tumor stages; 48% (32%), 45% (25%), 25% (12%), and 35% (21%) for stages I to IV, respectively (Fig 3F).

### Construction of phylogenetic trees of tumors with protein losses

**Fig 4:**
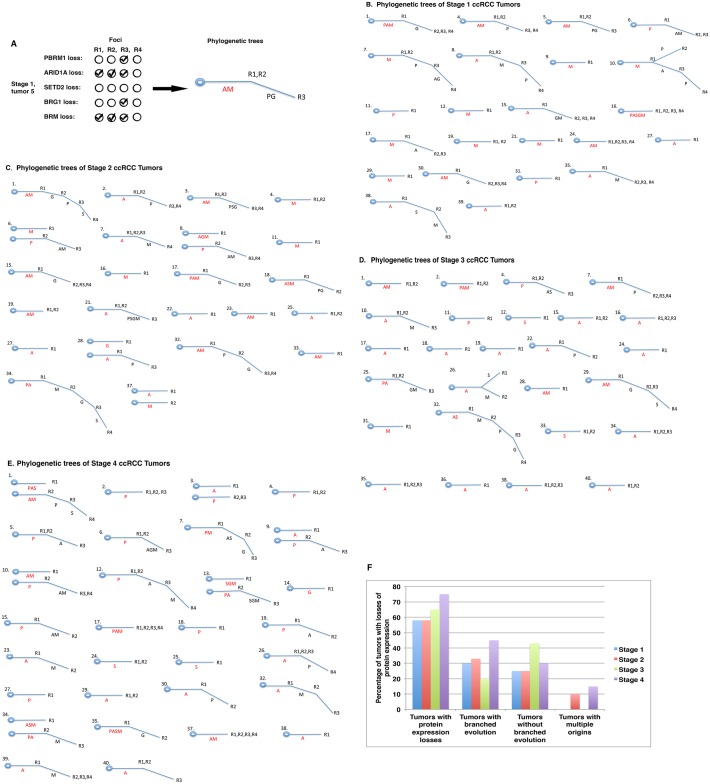
Phylogenetic trees of protein losses in ccRCC tumors. A) Depiction of the way in which a phylogenetic tree was constructed. A: ARID1A loss; M: BRM loss; P: PBRM1 loss, G: BRG12 loss; S: SETD2 loss. AM = losses of AIRD1A and BRM. PG = losses of PBRM1 and BRG1. R1, R2, R3 refer to the foci. Presentation of phylogenetic trees of stage I (B), stage II (C), stage III (D), and stage IV (E) ccRCCs. F) A summary of phylogenetic trees in tumors.

Ancestral relationships of different regions, e.g., quadrants, of a given tumor can be inferred by clonal ordering [26]. Subsequently, a phylogenetic tree can be constructed. If a molecular event happens very early during tumor evolution, it will be present in all or most regions of a tumor and it will be detected in most foci from the same tumor. Such an event is usually considered a truncal (early) change. Conversely, if a molecular event occurs late during tumor development, its presence will be limited to a small region of the same tumor, and it might not be detected at all or only detected in one or two foci that also have the truncal change(s). Such an event is considered a branch (late) change. For instance, in tumor 5 from our stage I group, losses of ARID1A and BRM were each detected in the first three foci, whereas losses of PBRM1 and BRG1 occurred only in the third focus (Fig 4A, *left*). In this case, losses of ARID1A and BRM were deemed to be truncal events, whereas losses of PBRM1 and BRG1 were considered branch events. Thus, a phylogenetic tree was built based on this interpretation (Fig 4A, *right*). Using this method we created phylogenetic trees of tumors with protein losses in tumor stage I (Fig 4B), stage II (Fig 4C), stage III (Fig 4D), and stage IV (Fig 4E). It is worth noting that these phylogenetic trees had different shapes. Some had only a trunk, e.g., stage I tumors 11, 12 and 16 with loss of one or more markers (Fig 4B). Other tumors had branches, e.g., stage II tumors 1, 2, 3, and 6 (Fig 4C). Occasionally, tumor branches bifurcated, e.g., stage III tumor 26 (Fig 4D). Infrequently, tumors displayed two trunks instead of one, with examples being stage IV tumors 1, 3, 9, 10, 13, and 34 (Fig 4E). In summary, 58%, 58%, 65% and 75% of tumors showed losses of at least one marker in tumor stages I, II, III and IV, respectively. From stage I to stage IV, there was no clear trend of a selection for tumors with or without branches. In tumor stages II and IV, a small percentage of cases exhibited two trunks instead of one (Fig 4F).

ITH and phylogenetic trees are traditionally examined based on DNA mutations in a limited number of samples. Our investigation clearly shows that IHC is able to accurately reveal ITH in ccRCCs at the protein levels on a larger scale. This afforded us the opportunity to statistically analyze the association between ITH and various biological or clinical parameters, which will be described elsewhere.

### Co-losses of protein markers in both tumors and foci

**Fig 5:**
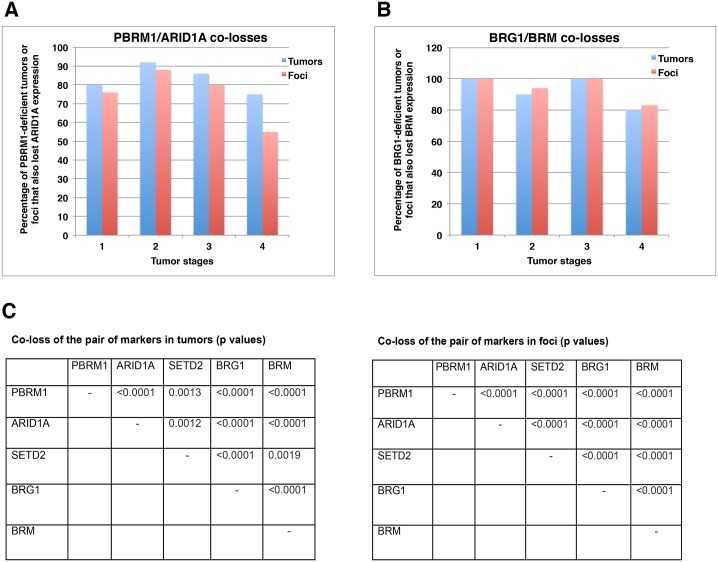
Co-losses of protein markers in both tumor and foci. A) Co-loss of ARID1A and PBRM1 expression in tumors/foci. B) Co-loss of BRM and BRG1 expression in tumors/foci. C) Statistical analysis of co-losses of the different protein markers in ccRCC tumors and foci. Co-losses of any two parameters considered here were found to be non-random.

While the protein expression losses were tallied on foci and tumors, one conspicuous observation was made: many markers were lost in the same foci and tumors. For instance, in tumors with PBRM1 loss, 75% to 90% of the same tumors also had co-loss of ARID1A (Fig 5A). This happened in all the tumor stages, and a similar pattern was observed when an individual focus was considered (Fig 5A). Similarly, 80% to 100% of tumors or foci with BRG1 loss also had co-loss of BRM (Fig 5B). Statistical analysis of all the possible pairs revealed that PBRM1/ARID1A, PBRM1/SETD2, PBRM1/BRG1, PBRM1/BRM, ARID1A/SETD2, ARID1A/BRG1, ARID1A/BRM, SETD2/BRG1, SETD2/BRM, BRG1/BRM, all had statistically significant co-losses in both tumors and foci (the biggest 2-sided *p* values was 0.0019, based on Fisher’s exact test) (Fig 5C). The original comparisons are shown in S2 Table. Thus, in this set of ccRCC samples, losses of these markers were highly correlated with each other and did not occur randomly. In kidney cancer cells, these proteins did not rely on each other for protein expression, as the shRNA-mediated suppression of each mRNA encoding these proteins did not lead to a reduction in the expression of other proteins.

### ARID1A loss appears to have greater tumor-promoting effect than PBRM1 loss

**Fig 6:**
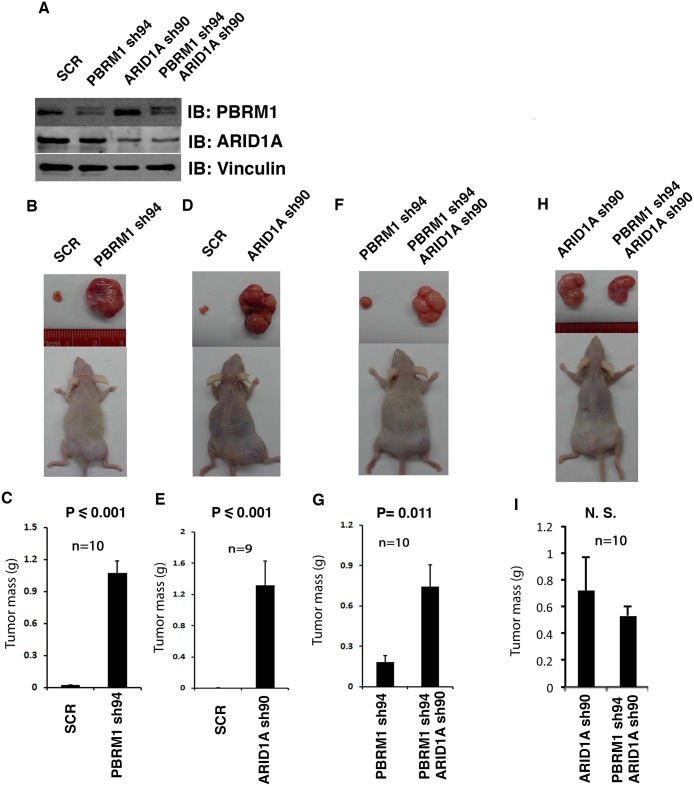
ARID1A loss seemed to have greater tumor-promoting effects than PBRM1 loss. A) Western blots showing suppression of PBRM1 and ARID1A expression. B, D, F, H) Representative photographs of excised tumors from tumor-bearing recipient mice. C, E, G, I) Summary of xenograft studies comparing tumor growth of the indicated cell lines. N.S.: not significant. n indicates the number of mice used in each experiment.

It is not known whether co-loss of these protein markers significantly impacts ccRCC tumor biology. To investigate this possibility, we next tested whether co-losses of PBRM1 and ARID1A affect tumor growth in a xenograft model when compared to single losses. We first verified that our shRNA constructs knocked down the expression of intended targets, as expected (Fig 6A). The same number of cells expressing either control (scrambled, SCR) or experimental shRNA were injected into opposite flanks of the same immunocompromised nude mice so that they shared a nearly identical physiological environment. As the durations of tumor growth for different groups usually differed, and the dominant tumor in the same mouse sometimes seemed to repress the growth of the other tumor, we think that only the comparisons between the pairs in the same mice are valid, while the cross-group comparisons, either of the same or different genotypes, are invalid. When PBRM1 expression was knocked down in 786-O VHL-deficient ccRCC cells, the tumors they generated were much larger than the ones from the control cells (Fig 6B and 6C). A similar result was observed when ARID1A was suppressed, suggesting that PBRM1 and ARID1A are two potent tumor suppressors that inhibit the rate of tumor growth (Fig 6D and 6E). Interestingly, when both PBRM1 and ARID1A were simultaneously knocked down in 786-O cells, much bigger tumors were seen than when only PBRM1 was suppressed (Fig 6F and 6G). However, when PBRM1 and ARID1A were both knocked down in 786-O cells, the tumors were not bigger than the ones with their ARID1A knocked down (Fig 6H and 6I). Thus ARID1A knockdown seemed to have a more potent tumor-promoting effect than PBRM1 knockdown. It is worth noting that the suppression of PBRM1 did not reduce the protein expression of ARID1A in 786-O cells (Fig 6A and S2 Fig), thus the co-loss of ARID1A in tumors with PBRM1 mutations is correlative, not a result of PBRM1 loss. ARID1A loss contributes to tumor growth significantly, but it is unlikely to be the major mechanism of tumor suppression by PBRM1.

## Discussion

ITH presents an important challenge for the field of precision medicine. The goal of precision medicine is to treat patients with drugs that specifically attack the vulnerabilities conferred by the major driving mutations in a given cancer. For example, imatinib (Gleevec) is used to treat chronic myeloid leukemia [27] to block the BCR-ABL fusion kinase and gastrointestinal stromal tumors [28] to block hyperactive, mutant KIT, Gefitinib is used to treat non-small cell lung cancer patients with tumors exhibiting mutant EGFR [29], and Vemurafenib to treat melanoma because it is a potent inhibitor of hyperactive, mutant BRAF [30]. Because of the specificity of these drugs, they would mostly spare the genetically normal cells of the body, so usually the side effects are mild. Unfortunately, they would also have little effect on a subpopulation of malignant cells of a tumor that do not have mutations on the target genes. ITH could mean that a subpopulation of such drug-resistant cancer cells would naturally exist in most tumors, and over time these cells would dominate and render the targeted therapies useless. Alternatively, even if all the cancer cells have mutations in the target genes, ITH would mean a fraction of cancer cells also have mutations in other genes or epigenetic changes that would render the cells resistant to the targeted therapies [31]. Over time, drug sensitive cancer cells would be killed off, while the drug-resistant subpopulation expands. ITH can also pose a challenge for animal models of different human cancers. It is difficult to genetically engineer model organisms with half a dozen or more mutations or to induce tumors in which some mutations are only present in a fraction of the neoplasm. Despite their value in studying cancer, this is one of the reasons why animal models do not completely recapitulate the setting in human disease.

ITH is very prevalent in various human malignancies, and it potentially has a huge impact on how we mange and treat cancers. However, our understanding of ITH is still quite limited. DNA sequencing for mutations, either by whole exome scanning or by use of a targeted panel, has emerged as an excellent tool to study ITH. It provides a very accurate representation of ITH in tumors, and the data quality is very high. In the case of whole exome sequencing, profiles of different portions of a given tumor can provide a comprehensive view of the heterogeneity within it. However, the drawback of this approach is that DNA sequencing still tends to be expensive and labor intensive, so reports of these studies usually consist of a dozen or so tumors, even when only a panel of genes is sequenced. Such a low number of samples do not provide enough statistical power to establish an association between phylogenetic trees with clinical parameters such as patient survival and response to drugs. Regional ploidy analysis can reveal ITH but is lacking in other details, while karyotypic and chromosome microarray analysis are not convenient for large-scale analysis because of cost and/or labor. Thus, we sought to address this problem by investigating whether we could reveal ITH by performing IHC on TMAs, a very mature technique that can simultaneously analyze a large number of samples at a relatively low cost.

However, a major concern for IHC is signal specificity, i.e., are the positive signals coming from the protein of interest or cross-reactivating proteins? In the case of loss of expression, did the IHC simply fail on the sample? Thus, the choice of antibodies to be used is crucial to the credibility of IHC study. We chose antibodies that have been validated in other studies as well as in our own hands. A key method to validate IHC signals is to compare them with DNA mutation profiles in the same tumor samples. It has been found that tumor samples harboring DNA mutations in a tumor suppressor gene also tend to lose expression of the encoded protein when examined by IHC, so we chose antibodies that were shown to pass this test where possible. We further examined the specificity of the chosen antibodies using shRNA and immunoblotting. Since these antibodies passed these tests, we are confident that the IHC signals are specific.

Our IHC analyses revealed that ITH is prevalent in ccRCC. In most cases, lost of expression of any given protein did not happen in all four foci from the same tumor (Fig 3A), similar to what has been reported in studies of DNA mutations in ccRCC. For some protein markers we examined, the incidence of loss of the expression was elevated in higher tumor stages, while with other markers, loss of expression was reduced in higher stages. With the expression loss data, we were able to construct phylogenetic trees of each individual tumor (Fig 4). The relatively large number of samples also ensured that we could find statistically significant correlations between protein expression losses and various clinical parameters, including disease-free survival, which will be presented elsewhere.

The close examination of the protein loss patterns revealed surprising co-losses of the markers (Fig 5), and our use of a xenograft model offered a method to evaluate the biological consequence of such losses (Fig 6). How mechanistically such combinations impact tumor behavior and drug response are clearly worthy of further investigation. In the renal cancer cell lines the suppression of one protein did not seem to impact the expression of other biomarkers we studied (Fig 6A, and S2 Fig), suggesting that the co-losses were likely created by biological pressures that led to further tumor development, instead of the co-dependence of each other for protein expression/stability. Such co-losses were clearly not recapitulated in the xenograft model, probably because it would take a long time for the secondary changes to occur in human tumors whereas the short time frame of the xenograft analysis did not allow that to happen.

Previous studies have shown that when BRG1 is deficient, cancer cells become dependent on the presence of BRM for survival [32, 33]. Consistent with this notion, we found that in 786-O kidney cancer cells efficient knockdown of both BRG1 and BRM could not be simultaneously achieved. However, concurrent losses of BRG1 and BRM were observed in SCCOHT tumor samples and isolated cancer cells [24]. It was also observed in cell lines from rhabdoid cancer, adrenal carcinoma and non-small cell lung cancer [34, 35]. Induction of BRM expression actually suppressed the growth of the rhabdoid cancer cells [34]. Thus, it appears that acute co-loss of BRG1 and BRM is not well tolerated by cultured cancer cells, but chronic co-losses occur in certain tumors and cancer cell lines. It is possible that some genetic or epigenetic alterations permit cancer cells to tolerate co-loss of BRG1 and BRM in these tumors.

DNA sequencing of multiple tumor regions provides a wealth of high quality data, but the current costs and labor involved make it difficult to apply to studies of ITH on a large scale. IHC is a technique that is relatively inexpensive to survey large number of samples if TMAs are constructed. It is worth pointing out that alterations detected by sequencing and IHC are not always consistent with each other. DNA mutations do not necessarily lead to changes in protein levels, and loss of protein expression certainly may arise for reasons other than DNA mutations. Proteins are the executors of the biological actions while the DNA is the blueprint. Protein expression changes are more nuanced and fluid while DNA mutations are more definitive. Thus, these approaches are mutually supportive and should both be employed for ITH studies.

ITH is a product of tumor evolution and can reflect adaptation to the microenvironment during the natural history of a neoplasm. ITH might also play a critical role in therapeutic efficacy and drug resistance. A multisite, dynamic survey of ITH within the same patients over time might elucidate how tumors evade treatments, and also provide clues about ways to modify treatments to manage a changing enemy effectively.

## Conclusion

IHC can successfully detect prevalent ITH at a large scale. We also observed striking co-losses of many markers. Using xenograft analysis, we provide evidence suggesting that ARID1A loss might be more potent in enhancing tumor growth than PBRM1 loss.

## Supporting Information

S1 Fig The original western blots for the validation of the antibodies used for IHC. (TIF)

S2 Fig Suppressions of PBRM1 did not affect the protein expression of ARID1A. Soluble lysates from 786-O kidney cancer cells stably expressing control shRNA or shRNA against *ARID1A*, *PBRM1*, *or JARID1C* were used for western blot analysis with indicated antibodies.
(TIF)

S1 Table The scores of the IHC signals of PBRM1, ARID1A, BRG1, BRM and SETD2 on each focus of the TMA. (XLS)

S2 Table Comparisons of the all the pairs of markers for potential co-losses in tumors and foci. Statistical analysis of co-losses of the different protein markers in ccRCC tumors and foci. 0 stands for expression loss and 1 for positive expression.
(XLSX)
