# Supplementary figures and images for: Immunohistochemistry Successfully Uncovers Intratumoral Heterogeneity and Widespread Co-Losses of Chromatin Regulators in Clear Cell Renal Cell Carcinoma
Source: PLoS One. 2016 Oct 20;11(10):e0164554. doi: 10.1371/journal.pone.0164554 (PMC5072613; doi:10.1371/journal.pone.0164554)

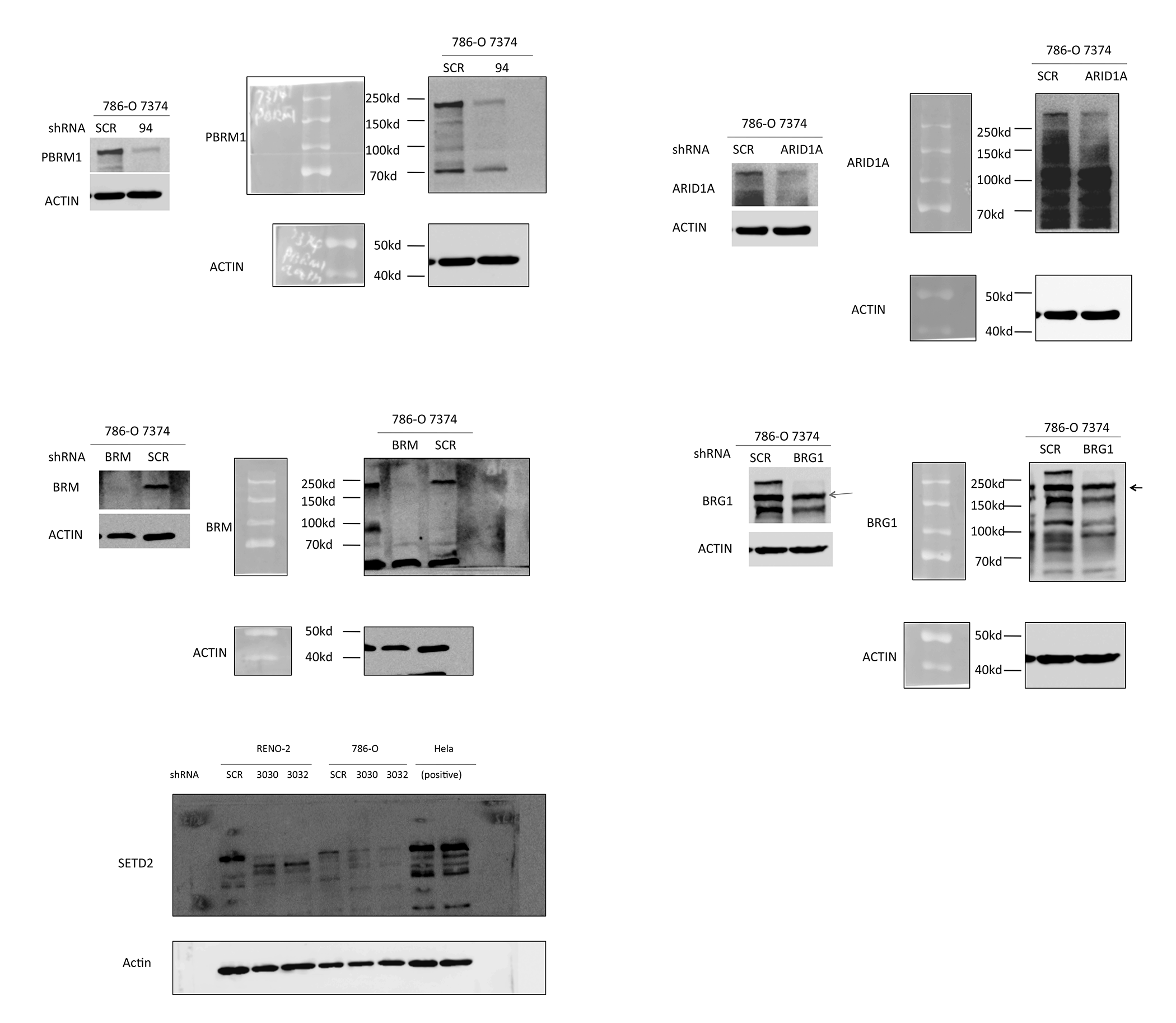

Supplement: S1 Fig — (TIF) [file pone.0164554.s001.tif]

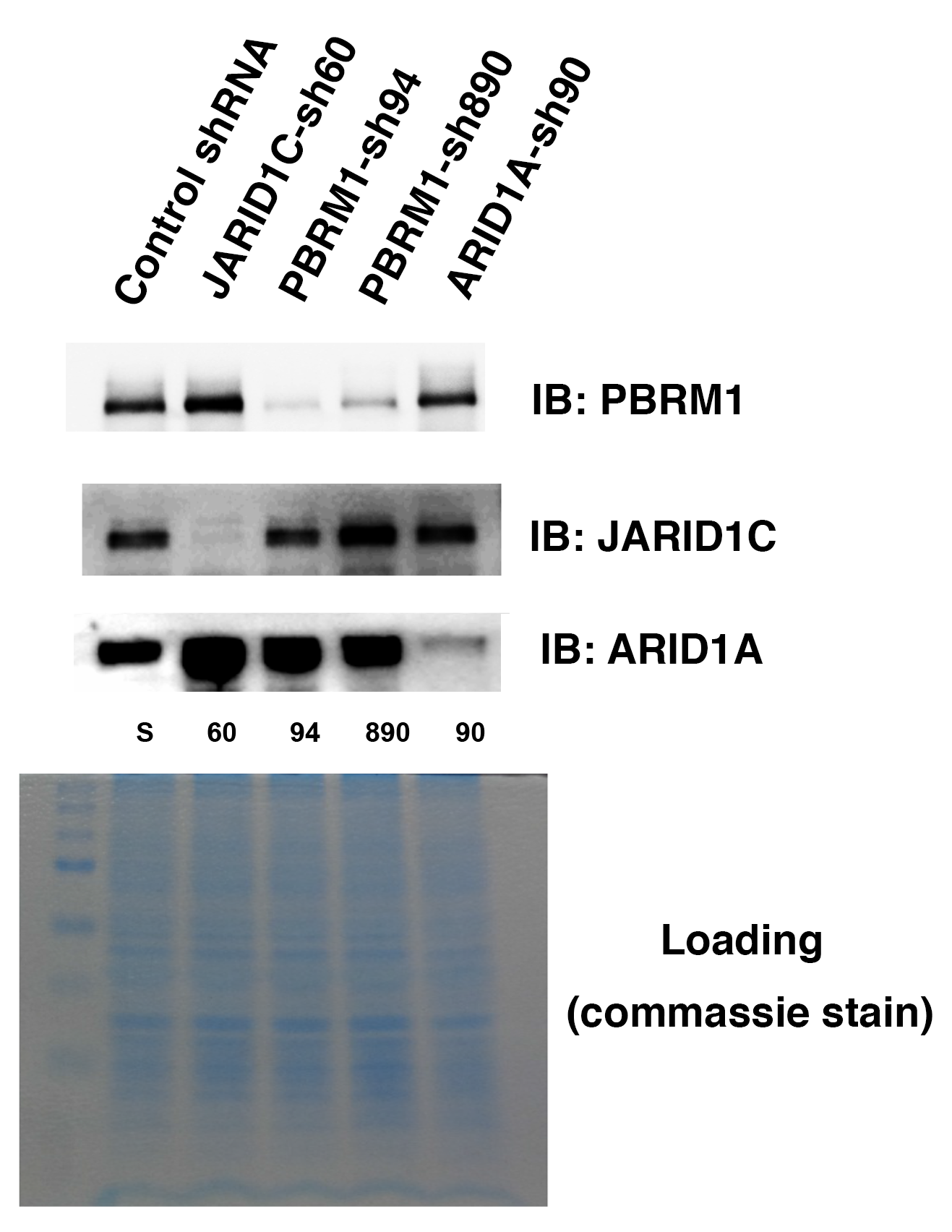

Supplement: S2 Fig — Soluble lysates from 786-O kidney cancer cells stably expressing control shRNA or shRNA against ARID1A, PBRM1, or JARID1C were used for western blot analysis with indicated antibodies. (TIF) [file pone.0164554.s002.tif]
